# A suspected case of rocuronium–sugammadex complex-induced anaphylactic shock after cesarean section

**DOI:** 10.1007/s00540-016-2280-4

**Published:** 2016-11-16

**Authors:** Masakazu Yamaoka, Miki Deguchi, Kiichiro Ninomiya, Toshiaki Kurasako, Mutsuko Matsumoto

**Affiliations:** 1 Department of Anesthesiology, Japanese Red Cross Society Himeji Hospital, 1-12-1, Shimoteno, Himeji, Japan; 2 Department of Allergy and Respiratory Medicine, Okayama University Hospital, Okayama, Japan

**Keywords:** Rocuronium, Sugammadex, Rocuronium–sugammadex complex, Anaphylaxis, Cesarean section

## Abstract

An anaphylactic reaction during a cesarean section occurs rarely, and rocuronium is thought to be one of the common agents causing perioperative anaphylaxis. Here we report an anaphylactic shock after cesarean section that is suggested to be induced by the rocuronium–sugammadex complex. A 36-year-old primigravida underwent an elective cesarean section under general anesthesia due to placenta previa. While the operation was completed uneventfully, she developed anaphylactic shock following sugammadex administration. She was successfully managed with rapid treatments. Serum tryptase level was significantly elevated. Although sugammadex was first suspected to be the causative agent, the result of intradermal skin tests with sugammadex were negative. Surprisingly, a subsequent intradermal test with undiluted rocuronium caused the patient to fall into a state of shock. Furthermore, a later skin-prick test with pre-mixed rocuronium–sugammadex complex also revealed a strong positive reaction, and a test with only rocuronium showed negative. We finally concluded that the rocuronium–sugammadex complex is the causative agent in this case. To the best of our knowledge, this is the first report suggesting anaphylaxis caused by the rocuronium–sugammadex complex. This case highlights the importance of appropriate examinations to determinate the pathogenesis of anaphylaxis in order to establish risk reduction strategies.

## Introduction

Anaphylactic reactions during cesarean sections rarely occur but can be fatal for both the mother and fetus. The incidence of anaphylaxis during general anesthesia has been estimated to be one case in 10,000–20,000 in the general population [1, 2]. It has also been reported that anaphylaxis during pregnancy occurs in approximately 3 cases per 100,000 deliveries [3]. A neuromuscular blocking agent, rocuronium, is thought to be a common agent causing perioperative anaphylaxis [4–6]. However, no case of anaphylaxis caused by the rocuronium–sugammadex complex has been reported. We report a suspected case of rocuronium–sugammadex complex-induced anaphylactic shock after cesarean section. Written consent was obtained from the patient to publish this case report.

## Case report

**Fig. 1:**
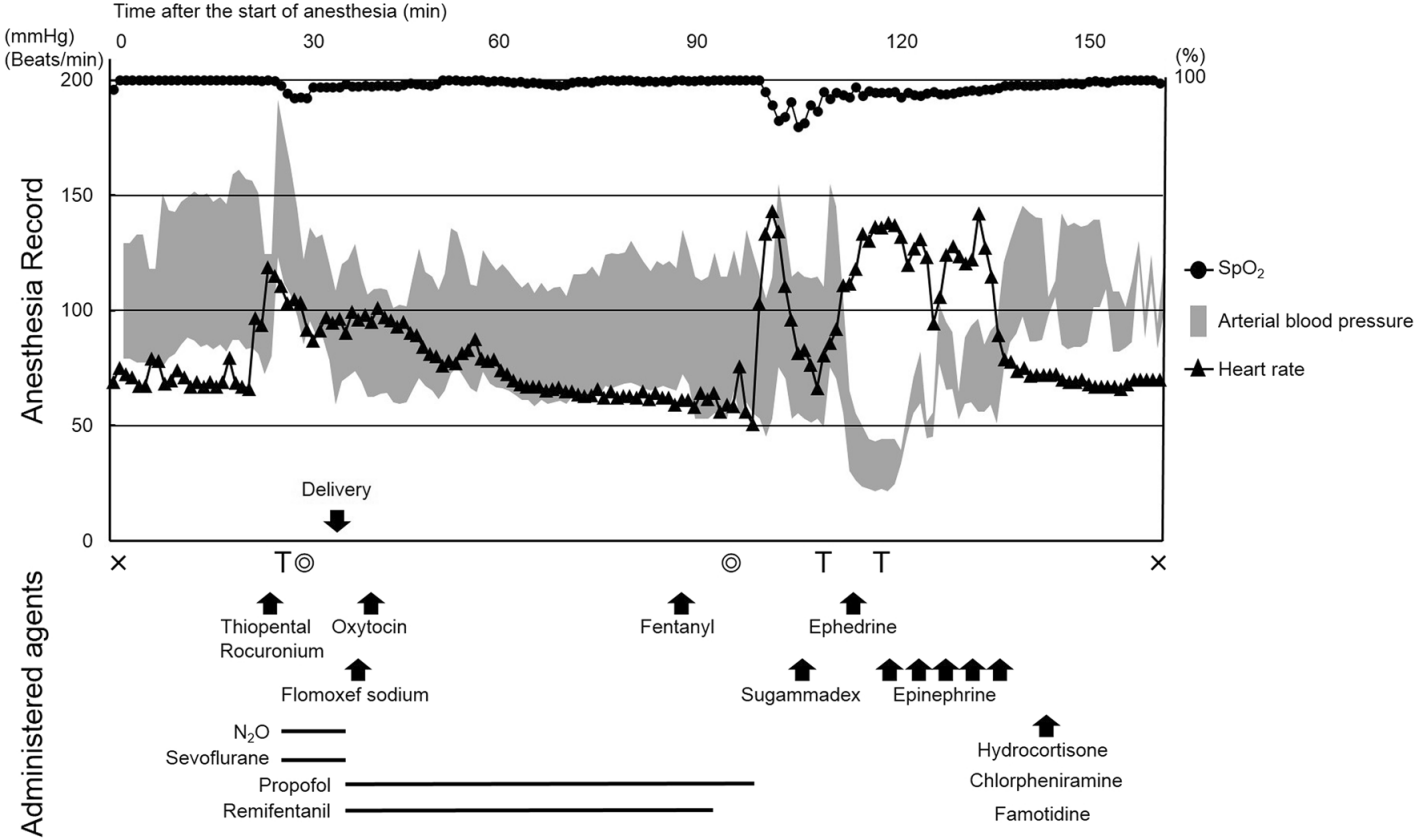
Anesthesia record. × Anesthesia start and end, *T* intubation and extubation, *concentric circles* operation start and end

A 36-year-old primigravida (body weight, 65 kg; height, 167 cm) with no history of drug allergy or surgery underwent an elective cesarean section because of placenta previa. General anesthesia was scheduled to secure the patient’s condition in case of massive bleeding. General anesthesia was induced with 350 mg thiopental and 70 mg rocuronium. After uneventful tracheal intubation, anesthesia was maintained with 1% sevoflurane and 50% nitrous oxide until delivery (Fig. 1). A neonate weighing 2774 g was delivered with Apgar scores of 8 and 9 at 1 and 5 min, respectively. After delivery, anesthesia was changed to total intravenous anesthesia with propofol, remifentanil, and supplemental bolus fentanyl. Immediately after ligation of the placenta, 1 g flomoxef sodium was intravenously infused to prevent surgical site infection and 5 U oxytocin was slowly infused intravenously 5 min after delivery. A final bolus of 200 μg fentanyl was infused approximately 15 min prior to the end of the operation. No additional rocuronium injection was required throughout the operation. The uneventful operation was completed in 66 min. The patient woke up approximately 10 min after the completion of the operation, and extubation was performed immediately following infusion of 200 mg sugammadex. Immediately after extubation, systolic arterial blood pressure suddenly fell to less than 40 mmHg, and the low level persisted despite fluid infusion and ephedrine administration. The patient complained of dyspnea and subsequently became unconscious. Manual mask ventilation and reintubation were successfully performed without rocuronium. Subsequently, her entire body became flushed, and transthoracic echocardiography revealed left ventricular collapse with no right ventricle dilatation, pericardial effusion, or aortic dissection. Arterial blood gas analysis revealed hemoconcentration compared with that at the beginning of the operation. Hemoglobin concentration was elevated from 10.9 to 16.1 g/dl, and hematocrit was elevated from 33.7 to 49.3%. We suspected a severe allergic reaction and continued shock treatment. Large amounts of intravascular fluids, repeat intravenous bolus of 0.1 mg epinephrine, and intravenous doses of 100 mg hydrocortisone, 20 mg famotidine, and 5 mg chlorpheniramine maleate were administered. After approximately 20 min, systolic arterial blood pressure had improved to over 100 mmHg and heart rate was normalized. No further anaphylactic reactions or other complications occurred. Mother and child were discharged on the seventh postoperative day. The results of blood tests revealed that the serum tryptase level at the time of the event was significantly elevated to 21.5 μg/l (normal range 2.1–9.0 μg/l) followed by a rapid decrease to 1.7 μg/l in a period of 24 h, strongly suggesting an anaphylactic reaction [7]. There was no significant increase in the serum histamine level (0.32 ng/ml, normal range 0.15–1.23 ng/ml).

We initially suspected the causative agent to be sugammadex because cardiac collapse occurred immediately after its administration. The patient was readmitted to our hospital 4 weeks after the event to undergo skin tests to detect the causative agent. The skin tests were performed by a dermatologist in the intensive care unit. First, a skin prick test of sugammadex was performed, resulting in no change. Then, intradermal skin tests with different concentrations were performed with 0.04 ml of each sample and produced 3–4 mm wheals. The diameter of wheal and flare was not increased by tests with sugammadex diluted 1:1,000, 1:100, or undiluted. Next, we performed an intradermal skin test using undiluted rocuronium (10 mg/ml) in consideration of the historical high incidence of rocuronium-induced anaphylaxis. This test was performed 40 min after the sugammadex tests. Surprisingly, 2 min after rocuronium administration, her vital signs rapidly progressed to a shock state. Systolic arterial blood pressure fell from 140 to 70 mmHg and heart rate increased from 70 beats/min to 140 beats/min. Her entire body showed generalized redness and she became dyspneic. Suspecting a possible allergic reaction, we started rapid management with intravenous fluid infusion, oxygen, and epinephrine. Her cardiorespiratory condition fully recovered in 20 min and there was no recurrence of the anaphylactic reaction.

The results of the intradermal skin tests were inconsistent with our expectations that sugammadex would reveal positive. We speculated that the rocuronium–sugammadex complex was the possible causative agent of the anaphylactic reaction. Hence, additional skin tests were performed by allergy specialists at the university hospital. First, a skin prick test with histamine as a positive control was performed and revealed a positive reaction with enlarged wheal reaction (from 9 × 5 to 30 × 30 mm) and itching paresthesia. Then, skin prick tests with rocuronium–sugammadex complex of different concentrations were conducted by pre-mixing sugammadex and rocuronium in test tubes (1:1 volume ratio). The diameter of wheal and a flare was not changed by each test with the complex diluted 1:1,000 and 1:100. However, a markedly positive persistent flare and enlarged wheal response with itching paresthesia was recorded at the test site with 1:10 diluted complex (from 8 × 6 to 35 × 24 mm), which was comparable to the histamine positive control. No subsequent intradermal test with the complex was conducted because even a skin prick test was positive. Two weeks later, a skin prick test with undiluted rocuronium was performed, resulting in no change. An additional intradermal test with undiluted rocuronium revealed enlarged wheal, but it was not accompanied by itching paresthesia and change of vital signs. Based on the results of the skin tests, we ultimately concluded that the rocuronium–sugammadex complex was the causative agent of the anaphylactic reaction.

## Discussion

In our patient, an anaphylactic shock occurred following a single intravenous dose of 200 mg sugammadex immediately after cesarean section. Considering the timing of the reaction, sugammadex was first suspected to be the causative agent. There are some case reports on sugammadex-induced anaphylaxis [8, 9]. Sugammadex directly encapsulates rocuronium to make it ineffective. It has recently been shown that sugammadex can be used for treatment of rocuronium-induced anaphylaxis by direct encapsulation [10, 11]. In contrast, to our knowledge, there has been no clinical report showing anaphylaxis caused by the rocuronium–sugammadex complex, although it has been suggested that this complex may provoke an allergic response [12]. It is surprising that the rocuronium–sugammadex complex can express new antigenicity even if rocuronium and sugammadex separately have no antigenicity. It is still controversial how the rocuronium–sugammadex complex formation can give rise to immunological change and allergenic behavior.

We consider that the reaction of the intradermal test with undiluted rocuronium at the university hospital, which revealed enlarged wheal without itching paresthesia, was false-positive. It was reported that rocuronium needs to be diluted at least 100-fold before skin tests to confirm hypersensitivity and to prevent false-positive responses [12, 13]. We believe our first test with undiluted rocuronium caused the patient to fall into a state of shock induced by formation of a rocuronium–sugammadex complex.

The combination of clinical, biochemical, and direct skin tests will enable the culprit agent to be identified. Some in vitro tools such as a histamine release test, flow-cytometric analysis of activated basophils, and a specific IgE assay have been proposed for clinical practice, but they are not universally available and are not as well established as skin tests [14, 15]. These in vitro tests may reinforce the hypothesis that the rocuronium–sugammadex complex provides allergic potency in this case.

It is important for anesthesiologists to identify the causative drug of perioperative anaphylaxis to prevent recurrence. Physicians often believe that the agent administered just prior to the event is the culprit allergen, without any subsequent examination. However, we should keep in mind that the suspected agent might be different from the true causative drug [16]. An incorrect speculation may place the patient at risk of exposure to the true allergen or cause unnecessary avoidance of a harmless effective drug. If we had not pursued the cause of the allergic reaction, this case would have been mistaken as a patient allergic to sugammadex. There may be patients allergic to the rocuronium–sugammadex complex who are considered to be allergic to sugammadex.

Should this patient undergo another general anesthesia, sugammadex must not be administered to reverse rocuronium. It might be advisable to avoid rocuronium and sugammadex altogether.

In conclusion, we experienced a case of anaphylaxis during a cesarean section that was suspected to have been induced by the rocuronium–sugammadex complex. It is important to determine the pathogenesis of anaphylaxis by appropriate examinations to establish optimal risk reduction strategies and to prevent recurrence.
